# Multi-scale characterization and mechanistic comparison of acid–alkali pretreatment during cell wall deconstruction of *Achnatherum splendens*

**DOI:** 10.1186/s40643-026-01097-2

**Published:** 2026-08-18

**Authors:** Zhennan He, Guolin Yang, Siyi Wang, Yuanyuan Jing, Fengqin Gao

**Affiliations:** 1 Grassland Research Institute, CAAS, Hohhot, 010010 Inner Mongolia China; 2 College of Grassland Science, Qingdao Agricultural University, Chengyang Campus, Qingdao, 266000 Shandong China

**Keywords:** *Achnatherum splendens*, Dilute sulfuric acid, Sodium hydroxide, Cellulose crystallinity, Lignocellulose

## Abstract

*Achnatherum splendens* was used as a high-lignin herbaceous feedstock to compare the effects of dilute sulfuric acid and sodium hydroxide pretreatments on component degradation and cell wall structure. Chemical composition analysis, X-ray diffraction (XRD), Fourier transform infrared spectroscopy (FTIR), and scanning electron microscopy (SEM) were used for multi-scale characterization. The results showed that dilute sulfuric acid pretreatment mainly promoted hemicellulose degradation, with a hemicellulose degradation rate of 79.2% under 1.5% H_2_SO_4_, while lignin dissolution remained limited at 20.5%. In contrast, NaOH pretreatment showed stronger lignin removal and fiber separation, with lignin dissolution increasing to 44.8% at 4% NaOH. Both pretreatments increased cellulose crystallinity from 47.0% to between 53.4 and 60.8%, whereas crystallite size showed no significant change, indicating that the crystalline cellulose domains were largely preserved. FTIR results showed spectral inconsistencies after acid treatment, including increased absorbance at 1510 cm^−1^ and limited reduction at 1730 cm^−1^, which may be associated with pseudo-lignin-related interference. SEM observations showed that acid treatment generated pores and cracks, whereas alkali treatment resulted in more obvious fiber bundle separation and surface cleaning. These results indicate that acid and alkali pretreatments have complementary effects on the deconstruction of *Achnatherum splendens*. The findings provide structural-level evidence for pretreatment selection and optimization of high-lignin energy grasses.

**Graphical abstract:**

**Figure:**
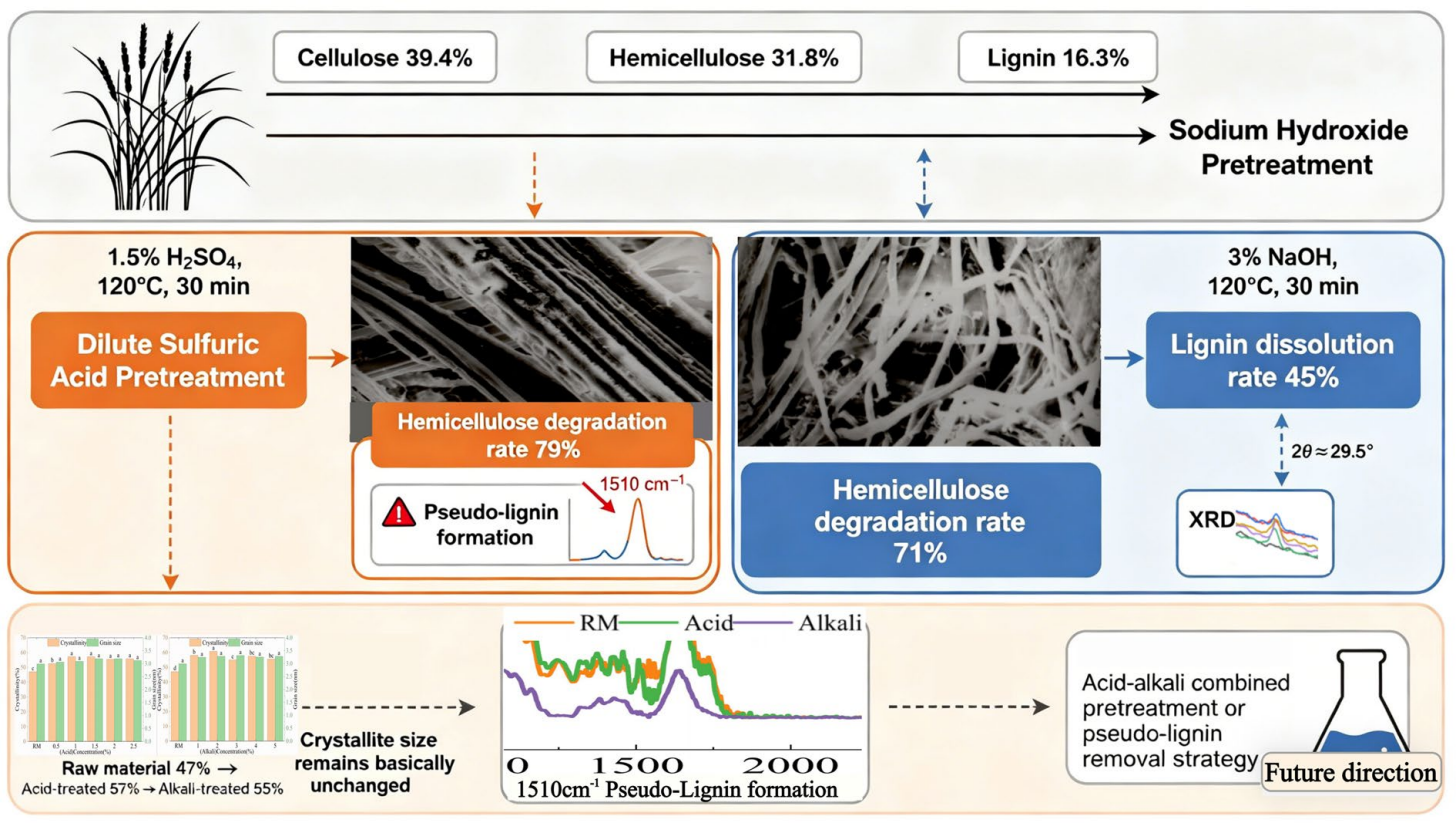

## Introduction

Lignocellulosic biomass is an important renewable resource for biofuels, biochemicals, and biorefinery applications. However, its efficient conversion is limited by the recalcitrant structure of plant cell walls, where cellulose, hemicellulose, and lignin are tightly associated through hydrogen bonding and covalent linkages. Pretreatment is therefore a key step for disrupting this structure and improving the accessibility of cellulose-rich fractions (Nair and Verma 2025).

Among chemical pretreatment methods, dilute sulfuric acid (H_2_SO_4_) and sodium hydroxide (NaOH) pretreatments are widely used because of their distinct deconstruction mechanisms (Behera et al. 2014). Dilute acid pretreatment preferentially hydrolyzes hemicellulose and increases cell wall porosity, whereas alkali pretreatment mainly promotes lignin dissolution and fiber swelling (Ji 2016; Miao et al. 2018). However, the effects of these two pretreatments vary depending on feedstock composition, especially lignin content. Therefore, a direct comparison of acid and alkali pretreatments using the same high-lignin feedstock is necessary to clarify their structural effects and process implications (Pari et al. 2025; Kululo et al. 2025).

*Achnatherum splendens* is a perennial grass species belonging to the Poaceae family and is widely distributed in the arid, semi-arid, and saline-alkali regions of northwest China. Owing to its strong tolerance to drought, cold, and saline-alkali stress, as well as its potential biomass productivity on marginal land, it may serve as a promising herbaceous feedstock for biomass utilization. Previous studies on *Achnatherum splendens* have mainly focused on chemical composition or general conversion potential, while systematic comparisons of acid and alkali pretreatment effects on crystallinity, functional groups, and microscopic morphology remain limited (Ni 2010; Tao et al. 2013; Ren et al. 2015). In particular, the structural responses of high-lignin *Achnatherum splendens* to different chemical pretreatments and possible FTIR spectral anomalies associated with acid pretreatment require further clarification.

In this study, dilute sulfuric acid and sodium hydroxide pretreatments were compared using *Achnatherum splendens* as the feedstock. Single-factor experiments were conducted to evaluate the effects of concentration, solid-to-liquid ratio, treatment time, and temperature on component degradation. XRD, FTIR, and SEM were further used to characterize changes in cellulose crystal structure, functional groups, and surface morphology. The objectives were to: (1) compare the degradation patterns of major components under acid and alkali pretreatments; (2) evaluate changes in cellulose crystallinity, crystallite size, and microstructure; (3) provide structural-level evidence for pretreatment strategy selection in high-lignin energy grasses.

## Materials and methods

### Materials

The samples of *Achnatherum splendens* were collected in September 2017 from the Xiaotangshan Base of the Beijing Academy of Agriculture and Forestry Sciences. After collection, the air-dried samples were cleaned to remove impurities, cut into 2–4 cm segments, thoroughly mixed, and subsequently stored in sealed bags before laboratory analysis. Before pretreatment, the samples were ground, passed through a 1 mm sieve, and then dried at 60–65 °C to determine the dry matter content.

### Pretreatment single-factor test

A single-factor experimental design was used to investigate the effects of four factors—concentration, solid-to-liquid ratio, treatment time, and temperature—on pretreatment efficiency. Each factor was set at five levels, and each treatment was performed in triplicate. The experimental design is shown in Table 1. For each treatment, 10 g of dried sample was mixed with the corresponding volume of H_2_SO_4_ or NaOH solution according to the predetermined solid-to-liquid ratio. The mixture was placed in a 500 mL Erlenmeyer flask and treated in a vertical autoclave. After pretreatment, the solid and liquid phases were separated by filtration through gauze. The solid residues were washed with tap water until neutral and dried to constant weight at 65 °C for subsequent analysis.

**Table 1:** Single-factor experimental design for *Achnatherum splendens* pretreatment

	Category	Concentration (%)	Solid–liquid ratio (g·mL^−1^)	Time (min)	Temperature (°C)
Acid	Single factor level	0.5	1:6	15	80
		1	1:8	30	100
		1.5	1:10	45	110
		2	1:12	60	120
		2.5	1:14	90	125
	Fixed factor	1:8 g·mL^−1^, 30 min, 120 °C	1.5%, 30 min, 120 °C	1.5%, 1:8 g·mL^−1^, 120 °C	1.5%, 1:8 g·mL^−1^, 30 min
Alkali	Single factor level	1	1:6	15	80
		2	1:8	30	100
		3	1:10	45	110
		4	1:12	60	120
		5	1:14	90	125
	Fixed factor	1:8 g·mL^−1^, 30 min, 120 °C	2.0%, 30 min, 120 °C	2.0%, 1:8 g·mL^−1^, 120 °C	2.0%, 1:8 g·mL^−1^, 30 min

Because acid and alkali pretreatments have different effective concentration windows, FTIR and SEM analyses were conducted using representative conditions of 1.5% H_2_SO_4_ and 3% NaOH to illustrate typical structural effects of hemicellulose hydrolysis (acid) and lignin removal, respectively.

### Chemical composition analysis

The chemical composition of raw and pretreated solid residues was determined using a FOSS semi-automatic fiber analyzer (FOSS, Denmark) according to the detergent fiber method of Van Soest et al. (1991). Before analysis, dried samples were ground and passed through a 1 mm sieve to ensure uniform particle size. A known mass of each sample was weighed accurately for detergent fiber analysis. Neutral detergent fiber (NDF) was first determined to estimate the total cell wall fraction, including cellulose, hemicellulose, lignin, and insoluble ash. Acid detergent fiber (ADF) was then determined to remove hemicellulose and obtain the cellulose- and lignin-rich fraction. The ADF residue was further treated with 72% H_2_SO_4_ to remove cellulose, and the remaining residue was ashed to correct for inorganic ash and determine the acid detergent lignin fraction. Each sample was analyzed in triplicate.

Cellulose and hemicellulose contents were calculated from the NDF, ADF, and lignin fractions (Table 2). The calculation formulas were as follows:

**Table 2:** Chemical composition of untreated *Achnatherum splendens*

Item	Content (%)
Cellulose	39.4
Hemicellulose	31.8
Lignin	16.3

1 $$\begin{array}{c}Lignin (\%) = \left(\frac{{M}_{ADF72}{ M}_{ash}}{{M}_{sample} DM}\right)\times 100\end{array}$$

2 $$\begin{array}{c}Cellulose (\%) = \left(\frac{{M}_{ADF-}{M}_{ADF72}}{{M}_{sample} DM}\right)\times 100\end{array}$$

3 $$\begin{array}{c}Hemicellulose (\%) = NDF (\%)-ADF (\%)\end{array}$$

In the formula, M_ADF_ denotes the mass of acid detergent fiber residue (g), while M_ADF72_ indicates the mass of ADF residue following treatment with 72% sulfuric acid (g). Additionally, M_ash_ represents the mass of crude ash (g) and M_sample_ signifies the initial mass of the sample (g). The variable DM corresponds to the dry matter content of the sample expressed as a percentage (%), whereas NDF (%) refers to the neutral detergent fiber content, and ADF (%) pertains to the acid detergent fiber content.

### X-ray diffraction analysis

The raw data were processed using OriginPro 2026 software. Initially, a multi-point baseline correction was applied, selecting 14 baseline points at 2θ values of 10.288036°, 11.05615212°, 12.255016°, 13.146633°, 13.267121°, 25.656380°, 27.635408°, 29.89156°, 31.912759°, 37.46428°, 40.886161°, 45.190622°, 49.510145°, and 49.898721°, where no diffraction peaks or primarily background signals were observed. The background was fitted using spline curves and subsequently subtracted. The diffraction patterns were then subjected to peak deconvolution using the Voigt function, which identified four crystalline peaks at approximately 2θ values of 14.6°, 16.0°, 22.5°, and 34.5°, corresponding to the (1–10), (110), (200), and (004) crystal planes of cellulose Iβ, along with one amorphous peak at approximately 2θ of 18°. This peak deconvolution was performed to determine the full width at half maximum (FWHM) of the (200) diffraction peak, which was utilized for calculating the crystallite size.

Crystallite size was calculated using the Scherrer equation (1918):

4 $$\begin{array}{c}D =\frac{K\lambda }{\beta cos\theta }\end{array}$$

In the equation, D represents crystallite size (nm), K is the Scherrer constant, λ denotes the X-ray wavelength, β is the full width at half maximum in radians, and θ is the diffraction angle. Since instrumental broadening was not corrected, the crystallite size values reported in this study are apparent values used only for relative comparison among treatments.

The crystallinity index was separately calculated using the Segal method (1959):

5 $$\begin{array}{c}CrI(\%)=\frac{{I}_{200}-{I}_{am}}{{I}_{200}}X100\end{array}$$

Here, I_200_ represents the maximum diffraction intensity at approximately 2θ ≈ 22.5°, while I_am_ represents the minimum diffraction intensity at approximately 2θ ≈ 18.0°. All samples were measured under the same instrumental conditions.

### Fourier transform infrared spectroscopy analysis

FTIR spectra of raw and pretreated samples were collected using a Tensor 27 spectrometer (Bruker Optik GmbH, Germany). Dried samples were prepared using the KBr pellet method, and spectra were recorded over the range of 4000–400 cm^−1^.

The spectra were exported in transmittance mode and subsequently converted to absorbance using OriginPro 2026. Baseline correction was applied before analysis. To reduce the influence of pellet thickness and sample concentration, semi-quantitative comparison was performed using absorbance ratios of characteristic peaks within the same spectrum.

### Scanning electron microscopy analysis

The surface morphology of the samples before and after pretreatment was observed using a scanning electron microscope (S-530, Hitachi, Japan). Dried samples were mounted on conductive adhesive, sputter-coated with gold, and examined at an accelerating voltage of 25 kV.

### Data analysis

Single-factor experimental data were analyzed by one-way ANOVA using SPSS 26.0. Differences were considered significant at *P* < 0.05. Figures were prepared using Origin 2026.

## Results

### Component degradation under dilute sulfuric acid pretreatment

**Fig. 1:**
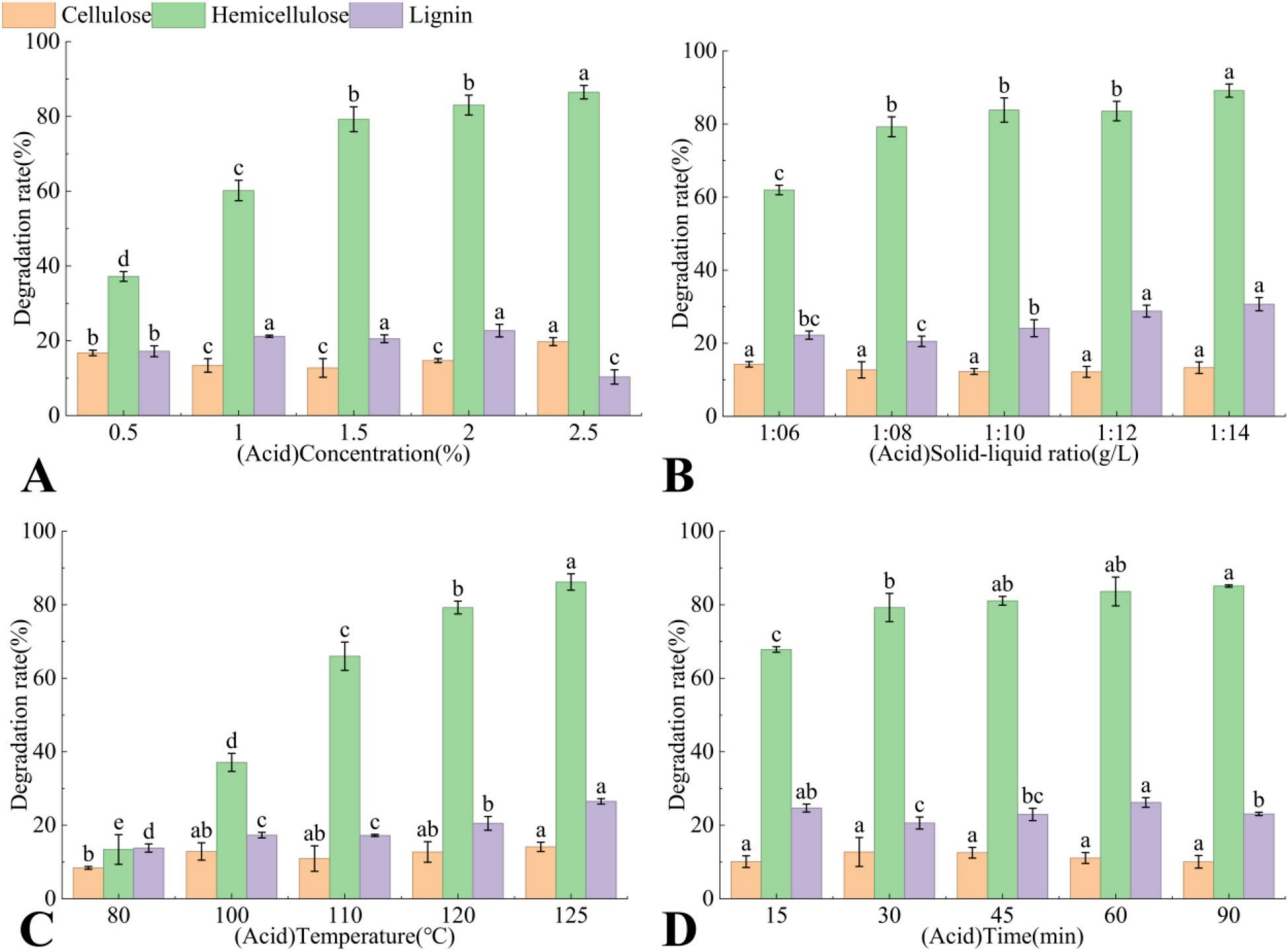
Effects of dilute sulfuric acid pretreatment conditions on component degradation of *Achnatherum splendens*. **A** Effect of H_2_SO_4_ concentration under a solid-to-liquid ratio of 1:8, 120 °C, and 30 min; **B** effect of solid-to-liquid ratio under 1.5% H_2_SO_4_, 120 °C, and 30 min; **C** effect of temperature under 1.5% H_2_SO_4_, a solid-to-liquid ratio of 1:8, and 30 min; and **D** effect of treatment time under 1.5% H_2_SO_4_, a solid-to-liquid ratio of 1:8, and 120 °C. Different lowercase letters indicate significant differences among treatments at *P* < 0.05

Under a solid-to-liquid ratio of 1:8, at 120 °C for 30 min, increasing the sulfuric acid concentration from 0.5 to 1.5% resulted in a significant rise in the hemicellulose degradation rate, from 37.2 to 79.2% (*P* < 0.05). Further increasing the concentration to 2.5% led to a plateau in the degradation rate. The cellulose degradation rate peaked at 19.8% with a sulfuric acid concentration of 2.5%, while the lignin dissolution rate did not show significant dose-dependency across the different concentrations (Fig. 1A). Under the conditions of 1.5% H_2_SO_4_, 120 °C, and 30 min, the hemicellulose degradation rate increased with the solid-to-liquid ratio, reaching 89.1% at a ratio of 1:14, significantly higher than in other treatment groups (*P* < 0.05). The cellulose degradation rate exhibited no significant differences among treatment groups, whereas the lignin dissolution rate was significantly higher at solid-to-liquid ratios of 1:12 and 1:14 (Fig. 1B). Under the conditions of 1.5% H_2_SO_4_, a 1:8 solid-to-liquid ratio, and 120 °C, the hemicellulose degradation rate reached 79.2% at 30 min, with no significant increase observed upon extending the duration to 60 min. The cellulose degradation rate showed no significant differences among the various time treatment groups, while the lignin dissolution rate peaked at 60 min (Fig. 1D). Temperature had the most substantial impact on hemicellulose degradation: as the temperature increased from 80 to 120 °C, the degradation rate rose from 13.4 to 79.2%, reaching 86.2% at 125 °C, with significant differences noted across all temperature gradients (*P* < 0.05). The dissolution rates of both cellulose and lignin attained their maximum values at 125 °C (Fig. 1C).

### Component degradation under sodium hydroxide pretreatment

**Fig. 2:**
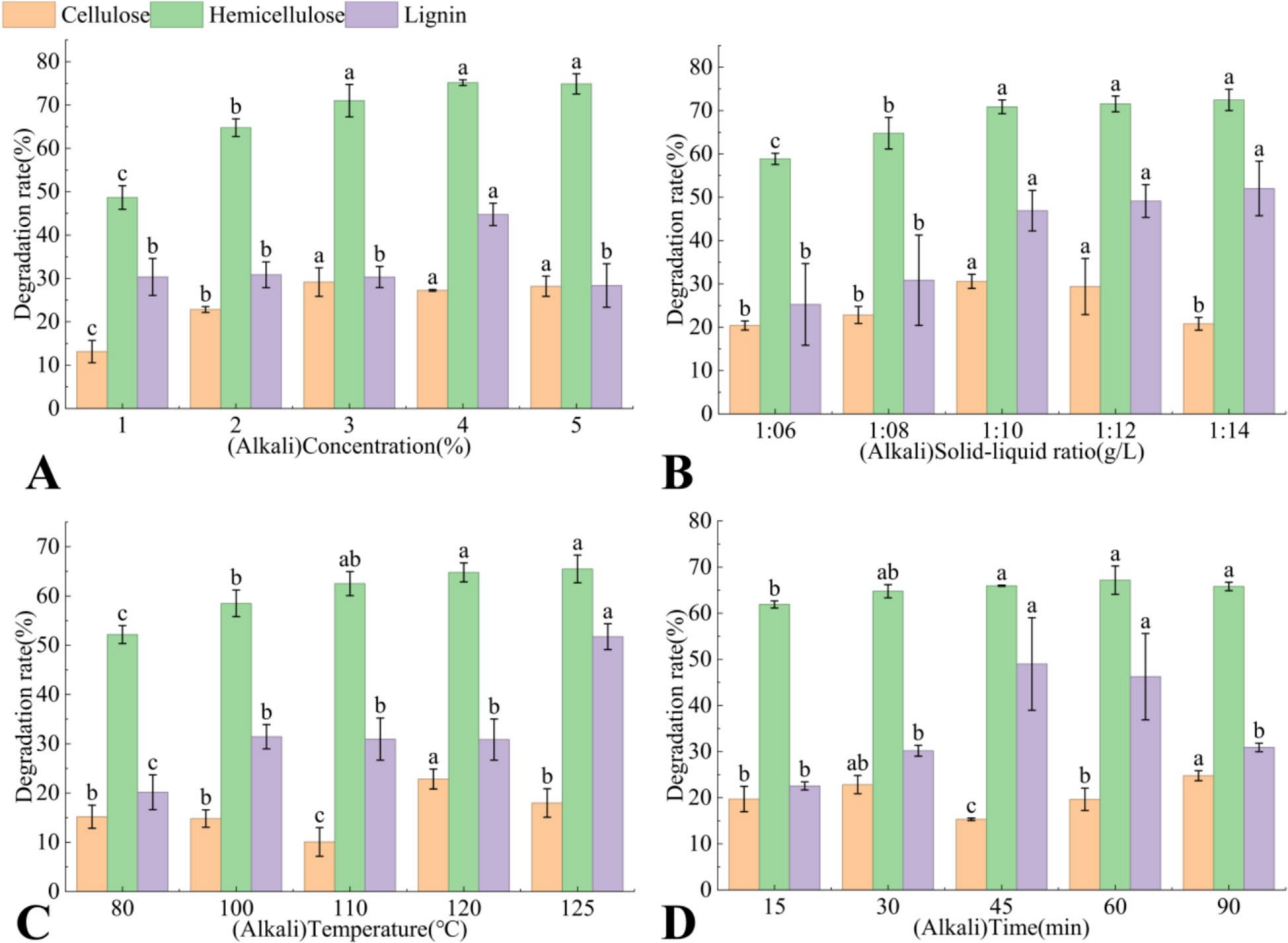
Effects of sodium hydroxide pretreatment conditions on component degradation of *Achnatherum splendens*. **A** Effect of NaOH concentration under a solid-to-liquid ratio of 1:8, 120 °C, and 30 min; **B** effect of solid-to-liquid ratio under 2% NaOH, 120 °C, and 30 min; **C** effect of temperature under 2% NaOH, a solid-to-liquid ratio of 1:8, and 30 min; and **D** effect of treatment time under 2% NaOH, a solid-to-liquid ratio of 1:8, and 120 °C. Different lowercase letters indicate significant differences among treatments at *P* < 0.05

Under a solid-to-liquid ratio of 1:8, at 120 °C for 30 min, the lignin dissolution rate remained stable between 30.3 and 30.9% as the NaOH concentration increased from 1 to 3%. A significant increase in the dissolution rate to 44.8% (*P* < 0.05) was observed at a concentration of 4%, but it subsequently decreased to 28.4% at 5% concentration. The hemicellulose degradation rate rose significantly to 71.0% (*P* < 0.05) at 3% NaOH concentration, with no significant increase noted within the 4–5% concentration range. The cellulose degradation rate exhibited an upward trend with increasing NaOH concentrations, where treatments at 3–5% concentrations demonstrated significantly higher degradation rates compared to those at 1–2% concentrations (Fig. 2A). Under conditions of 2% NaOH, 120 °C, and a 30-min duration, the lignin dissolution rate consistently increased with rising solid-to-liquid ratios, peaking at 52.0% at a ratio of 1:14. The hemicellulose degradation rate significantly reached 70.9% at a solid-to-liquid ratio of 1:10, with no significant increase thereafter. The cellulose degradation rate remained relatively high between solid-to-liquid ratios of 1:10 and 1:12 (Fig. 2B). Under the conditions of 2% NaOH, a solid-to-liquid ratio of 1:8, and 120 °C, the lignin dissolution rate significantly increased to 49.0% (*P* < 0.05) at 45 min but decreased to 30.6% at 90 min. The hemicellulose degradation rate showed no significant differences across varying treatment durations. The cellulose degradation rate significantly increased at 90 min (Fig. 2D). An increase in temperature from 80 to 125 °C resulted in a significant enhancement of the lignin dissolution rate from 20.2 to 51.7%, with the 125 °C treatment exhibiting a significantly higher dissolution rate compared to other temperature treatments (*P* < 0.05). The hemicellulose degradation rate was significantly higher at temperatures exceeding 110 °C compared to those at 80–100 °C. The cellulose degradation rate reached its lowest value of 10.1% at 110 °C (Fig. 2C).

### Effects of pretreatment on cellulose crystallinity and crystallite size

**Fig. 3:**
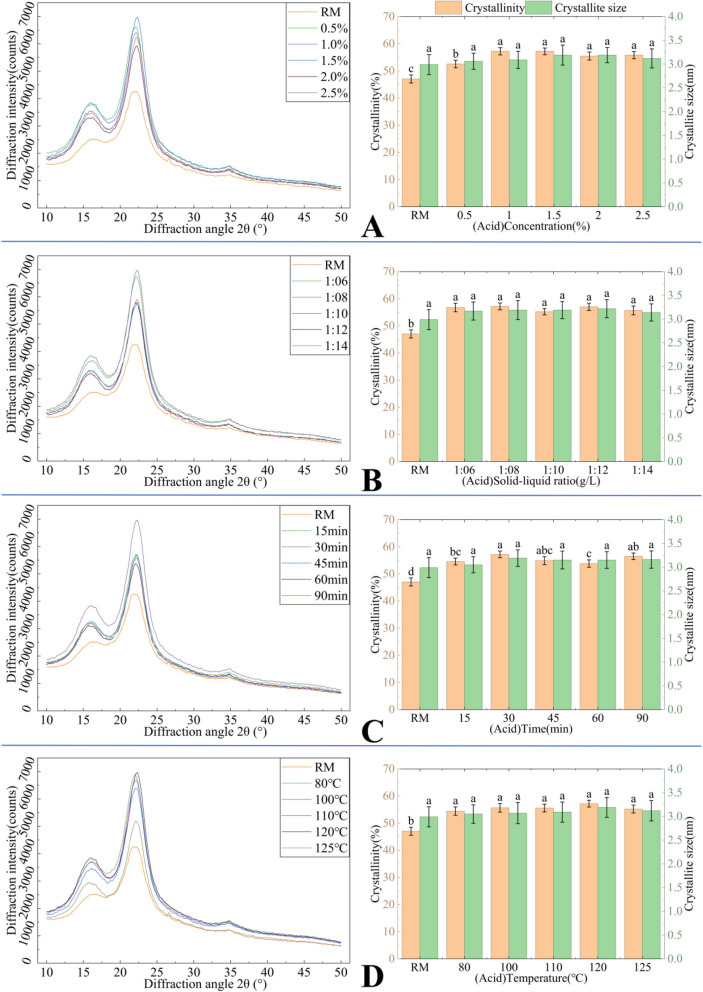
X‑ray diffraction patterns and crystallinity‑related parameters of Achnatherum splendens before and after dilute sulfuric acid pretreatment. **A** Effect of H2SO4 concentration; **B** effect of solid‑to‑liquid ratio; **C** effect of treatment temperature; **D** effect of treatment time. RM represents raw material without treatment. Lowercase letters indicate significant differences at *P* < 0.05

**Fig. 4:**
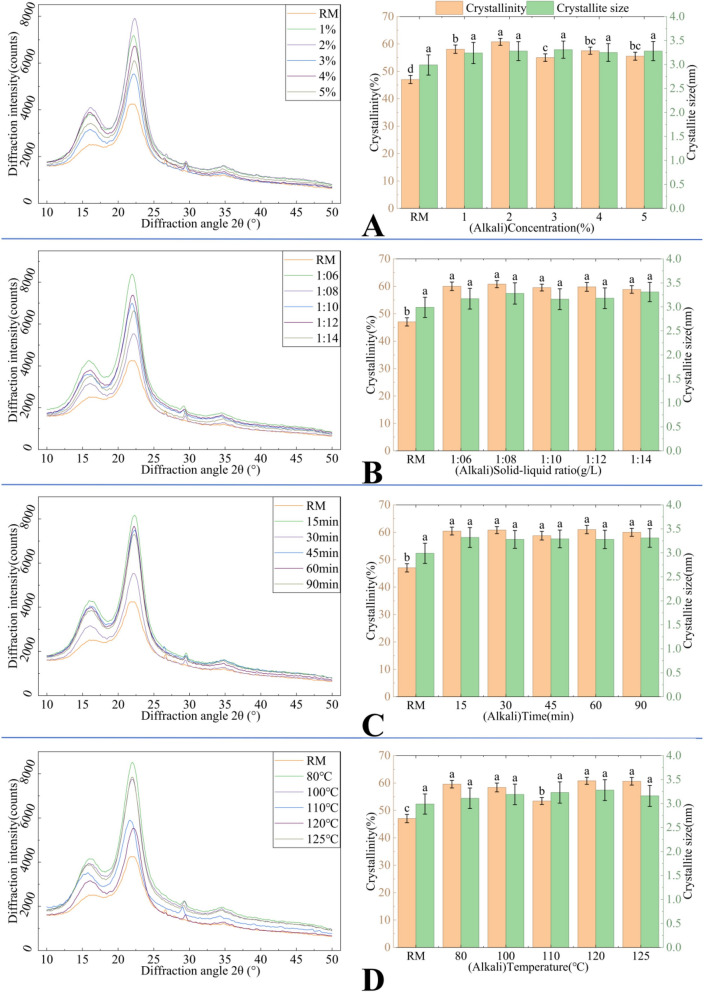
X‑ray diffraction patterns and crystallinity‑related parameters of Achnatherum splendens before and after sodium hydroxide pretreatment. **A** Effect of NaOH concentration; **B** effect of solid‑to‑liquid ratio; **C** effect of treatment temperature; ** D** effect of treatment time. RM represents untreated raw material. Different lowercase letters indicate significant differences among treatments at *P* < 0.05

The crystallinity of untreated *Achnatherum splendens* was measured at 47.0%. Following pretreatment with dilute sulfuric acid and sodium hydroxide, the crystallinity of all treated samples exhibited a significant increase (*P* < 0.05). Specifically, the crystallinity of acid-treated samples rose to between 55.2 and 57.2%, while alkali-treated samples increased to between 53.4 and 60.8% (Figs. 3 and 4). In the single-factor analysis of acid treatment, a concentration range of 1.0–1.5% yielded crystallinity values significantly higher than those observed at a 0.5% concentration. Additionally, a treatment duration of 30 min resulted in significantly higher crystallinity compared to 60 min; however, no significant differences were noted among varying solid–liquid ratios and temperatures. For alkali treatment, the highest crystallinity (60.8%) was achieved at a concentration of 2%, but it significantly decreased to 53.4% at 110 °C. Similarly, no significant differences were found among various solid-to-liquid ratios and treatment durations. The crystallite size of untreated *Achnatherum splendens* was 2.99 nm, while pretreated samples exhibited sizes ranging from 3.05 to 3.32 nm, with no significant differences across the various factor levels (*P* > 0.05). Notably, all NaOH-pretreated samples displayed a pronounced diffraction peak at 2θ ≈ 29.5°, which was absent in both the raw material and acid-treated samples.

### FTIR-based changes in functional groups after acid and alkali pretreatments

**Fig. 5:**
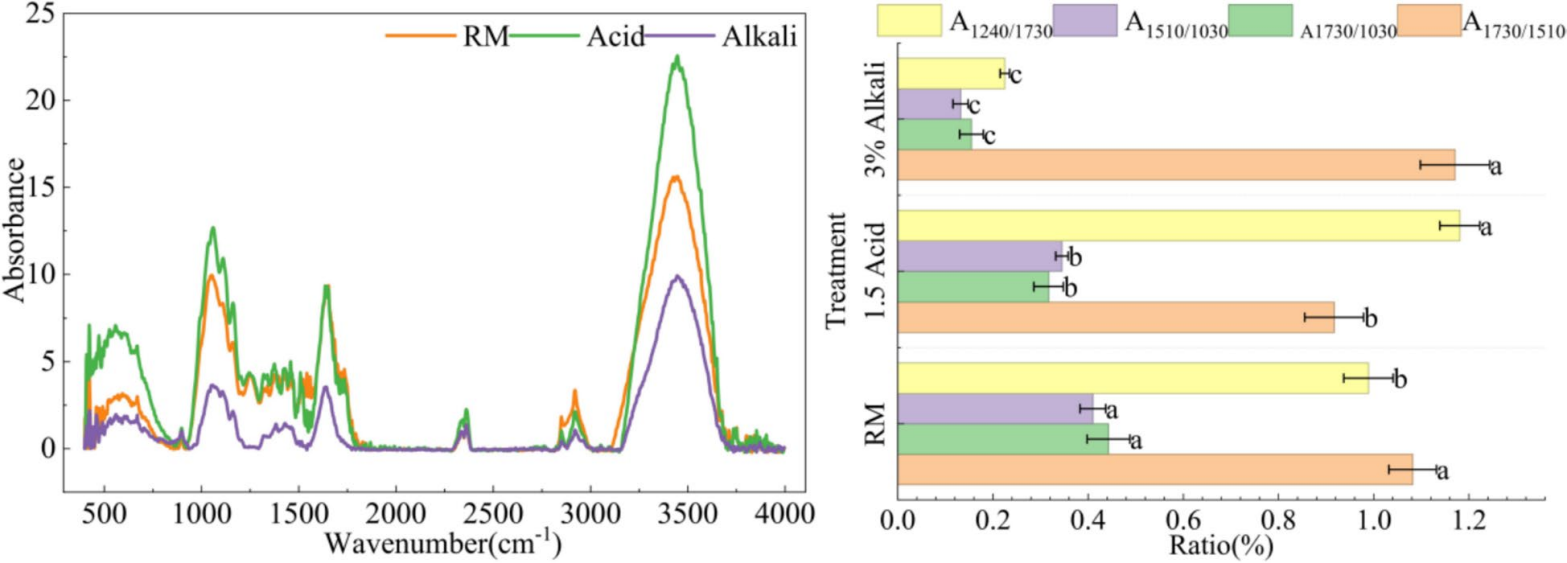
FTIR spectra of *Achnatherum splendens* raw material and acid/alkali pretreated samples. RM represents raw material without treatment. The absorbance ratios A_1240/A1730_, A_1510/A1030_, A_1730/A1030_, and _A1730/A1510_ were used for semi-quantitative comparison of functional group changes. Lowercase letters indicate significant differences at *P* < 0.05

After pretreatment with 1.5% H_2_SO_4_ at 120 °C for 30 min, the changes in absorbance at characteristic peaks were as follows: the absorbance at 1730 cm^−1^ decreased from 4.137 to 3.676, while it increased from 3.824 to 4.009 at 1510 cm^−1^, from 4.091 to 4.341 at 1240 cm^−1^, and significantly rose from 9.332 to 11.610 at 1030 cm^−1^, as well as from 0.676 to 1.178 at 898 cm^−1^. Meanwhile, the ratios A_1730_/A_1510_, A_1730_/A_1030_, and A_1510_/A_1030_ exhibited a decreasing trend, whereas the A_1240_/A_1730_ ratio increased from 0.989 to 1.181 (Fig. 5). Under the pretreatment conditions of 3% NaOH at 120 °C for 30 min, the absorbance changes at characteristic peaks became more pronounced: the absorbance at 1730 cm^−1^ sharply decreased to 0.493, at 1510 cm^−1^ to 0.421, at 1240 cm^−1^ to 0.111, and at 1030 cm^−1^ to 3.180, while it increased to 1.016 at 898 cm^−1^. Additionally, the ratios A_1730_/A_1030_, A_1510_/A_1030_, and A_1240_/A_1730_ all showed significant decreases. The comparative analysis of the effects of acid and alkali treatments revealed that after alkali treatment, the absorbance at 1730 cm^−1^, 1510 cm^−1^, and 1240 cm^−1^ decreased by 88%, 89%, and 97%, respectively. In contrast, acid treatment resulted in smaller reductions of these peaks, with the absorbance at 1510 cm^−1^ even showing an increase.

### SEM observation of surface morphology and fiber separation

**Fig. 6:**
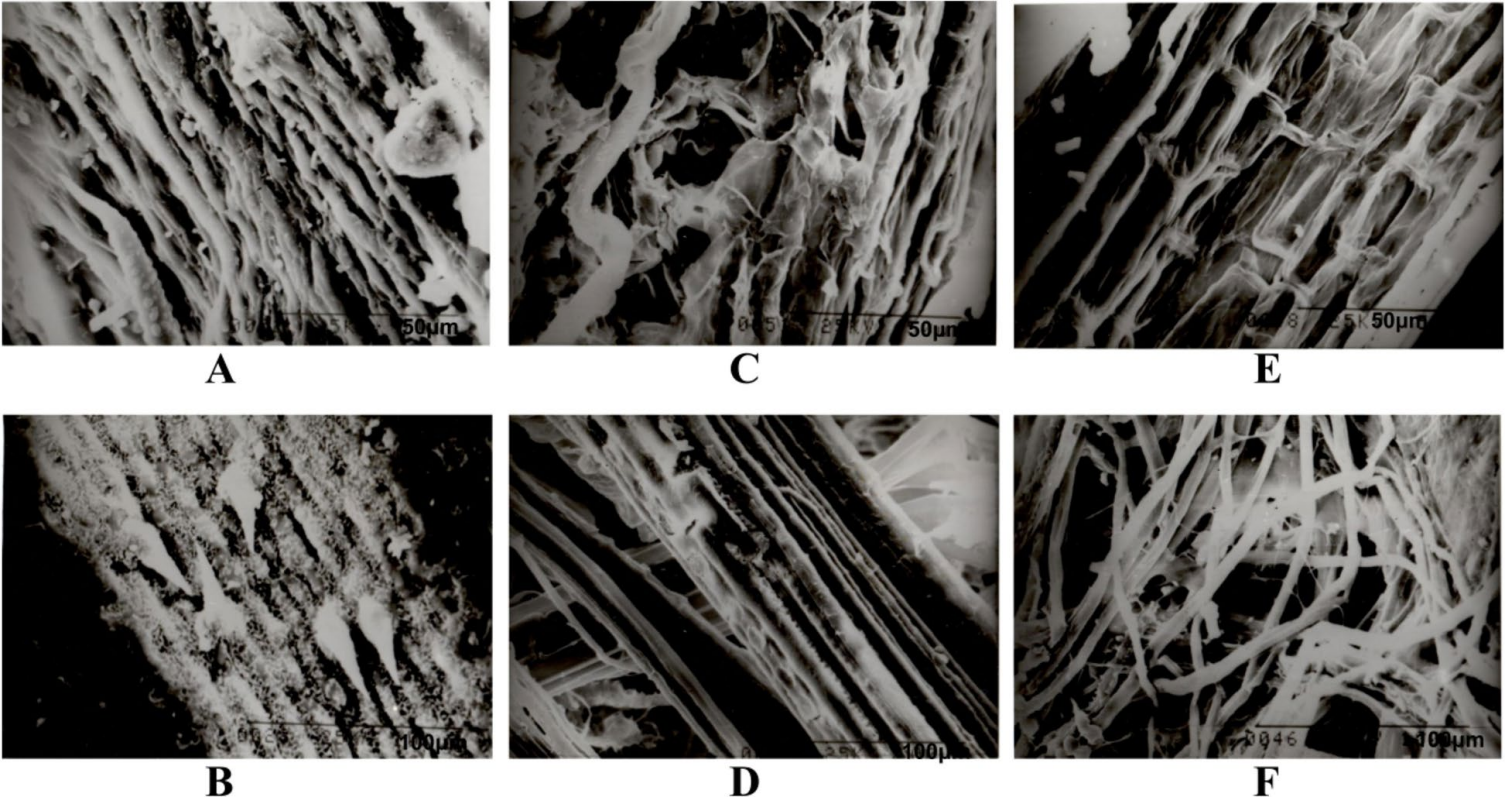
Scanning electron microscopy images of *Achnatherum splendens* before and after acid and alkali pretreatments. **A**, **B** Untreated raw material: **A** inner surface and **B** outer surface; **C**, **D** samples pretreated with 1.5% H_2_SO_4_ at 120 °C for 30 min: **C** inner surface and **D** outer surface; **E**, **F** samples pretreated with 3% NaOH at 120 °C for 30 min: **E** inner surface and **F** outer surface. Scale bars: 50 μm for A, C, and E; 100 μm for B, D, and F. Original scale bar labels were illegible and have been digitally redrawn for clarity without altering scale dimensions

The untreated inner surface of *Achnatherum splendens* (Fig. 6A) exhibited axially parallel-aligned fiber bundles characterized by a compact and continuous structure. The surface appeared smooth with minimal impurities, featuring extremely narrow inter-fiber gaps, thus presenting an overall dense and orderly arrangement. In contrast, the outer surface (Fig. 6B) displayed a rougher texture with distinct undulations, extensively covered by fine particulate matter—including siliceous, waxy, and cuticular fragments—that completely obscures the contours of the fiber bundles. After treatment with 1.5% H_2_SO_4_ at 120 °C for 30 min, the original parallel fiber bundle structure on the inner surface (Fig. 6C) was severely damaged, with fibers exhibiting twisting, breakage, and fragmentation, resulting in a loose flocculent or reticular structure. Numerous pores and gaps appeared between the fibers, and a small amount of granular or flaky residues remained on the surface. On the outer surface (Fig. 6D), most granular impurities were removed, revealing clearly visible fiber bundles that maintained their axial parallel arrangement. However, significant delamination and peeling occurred between the fiber bundles, with increased gaps, and a small amount of granular or flaky residues similarly remained on the surface. After treatment with 3% NaOH at 120 °C for 30 min, the inner surface (Fig. 6E) exhibited significant fibrillation of fiber bundles, characterized by tearing and branching on the fiber surfaces. Microfibrils were exposed, forming filamentous connections while maintaining a degree of axial alignment. However, the structure became more porous and less dense compared to the original inner surface. On the outer surface (Fig. 6F), the overlaying layer was completely removed, resulting in the complete separation of fiber bundles into individual fibers. These fibers became interwoven and twisted, creating an irregular network structure. Notably, the fiber diameter decreased significantly, accompanied by observable swelling.

## Discussion

### Raw material characteristics and their influence on pretreatment difficulty

The raw *Achnatherum splendens* material utilized in this study exhibited a composition of 39.41% cellulose, 31.82% hemicellulose, and 16.29% lignin. In comparison to previously reported compositional data for *Achnatherum splendens*, the raw material analyzed in this study displayed distinct characteristics of “low cellulose and high lignin.” Ni (2010) documented that *Achnatherum splendens* from Zhangye, Gansu, contained 45.31% cellulose and only 9.59% lignin. Furthermore, the *Achnatherum splendens* materials examined by Tao et al. (2013) and Ren et al. (2015) also demonstrated relatively low lignin content. These compositional discrepancies may be attributed to variations in regional climate, soil conditions, and the genetic backgrounds of different varieties.

Lignin content is a critical factor influencing the recalcitrance of feedstocks (Himmel et al. 2007; DeMartini et al. 2013). Elevated lignin levels result in cellulose being encapsulated within a dense lignin–hemicellulose composite matrix, thereby increasing mass transfer resistance. Furthermore, alkaline pretreatment necessitates greater consumption of hydroxide ions (OH^−^) to effectively remove lignin (Silverstein et al. 2007). Recent studies indicate that while dilute acid pretreatment can effectively decompose hemicellulose, its impact on enhancing enzymatic hydrolysis yield is limited for feedstocks with high lignin content. Research by Zhang et al. (2025a) on sunflower stalk rind revealed that after dilute sulfuric acid pretreatment, the enzymatic hydrolysis yield increased only from 15.2 to 26.8%, remaining below 30%. In contrast, NaOH pretreatment can concurrently remove both hemicellulose and lignin, achieving an enzymatic hydrolysis yield of 96.7%. These literature results suggest that lignin can act as an important physical barrier during enzymatic hydrolysis, although enzymatic hydrolysis was not directly tested in the present study. The *Achnatherum splendens* utilized in this study possesses a relatively high lignin content (16.29%), suggesting that its natural recalcitrance may be greater than that of low-lignin *Achnatherum splendens* samples reported in the literature. Therefore, the pretreatment behavior observed here should be interpreted in relation to the relatively high lignin content of the feedstock.

### Mechanistic differences between dilute sulfuric acid and sodium hydroxide pretreatments

#### Core mechanisms of acid and alkali pretreatments

Chemical composition analysis revealed that dilute sulfuric acid pretreatment exhibited high selectivity for hemicellulose hydrolysis, while sodium hydroxide pretreatment was characterized by efficient lignin removal alongside significant hemicellulose dissolution. Dilute sulfuric acid catalyzes the hydrolysis of glycosidic bonds in hemicellulose through H₃O⁺, promoting its degradation and dissolution. In contrast, cellulose demonstrates relative inertness to acid due to its high crystallinity, while lignin exhibits resistance owing to the stability of aromatic ether bonds (Ji 2016). In this study, treatment with 1.5% H_2_SO_4_ achieved a hemicellulose degradation rate of 79.2%, whereas the lignin dissolution rate was only 20.5%. Gao et al. (2020a) reported that the hybrid Pennisetum exhibited a slightly higher hemicellulose degradation rate (85.73%) under similar conditions, which may be attributed to the higher lignin content in *Achnatherum splendens* that hinders acid mass transfer. Zhang et al. (2025a) further confirmed in sunflower stem bark that while dilute sulfuric acid effectively decomposes hemicellulose, most lignin remains (removal rate only about 20%), resulting in a limited increase in enzymatic hydrolysis yield from 15.2 to 26.8%. SEM observations were consistent with the aforementioned compositional changes at the morphological level. Following acid treatment, fibers on the inner surface (Fig. 6C) exhibited characteristics of twisting, fracturing, and fragmentation, resulting in the formation of loose flocculent or reticular structures, alongside a small amount of granular or flaky residues visible on the surface. On the outer surface (Fig. 6D), granular impurities were largely removed, revealing clearly visible fiber bundles characterized by delamination and peeling phenomena, while similarly displaying a small quantity of granular or flaky residues. These residues may include incompletely removed native lignin and condensed pseudo-lignin products generated during the acid treatment process. Gao et al. (2020a) also observed similar phenomena regarding fiber bundle exposure and residue retention in acid-treated hybrid Pennisetum. The study by Gao et al. (2022) demonstrated that hybrid Pennisetum exhibited tightly arranged cellulose bundles following H_2_SO_4_ pretreatment, with FTIR analysis indicating the disruption of ether/ester bonds between lignin and carbohydrates. Shi et al. (2025) found that although acid-treated soybean straw achieved a soluble sugar recovery rate of 270 mg/g, its enzymatic saccharification yield was significantly lower than that of alkali treatment (787 mg/g), further suggesting that lignin residues may restrict enzymatic hydrolysis, although enzymatic hydrolysis was not evaluated in this study.

Alkali treatment facilitates delignification and fiber swelling through the saponification of lignin–hemicellulose ester bonds by hydroxide ions (OH^−^) and the cleavage of lignin phenolic ether bonds (Hendriks and Zeeman 2009; Miao et al. 2018). In this study, the lignin dissolution rate reached 30.33%, while hemicellulose degradation achieved 71.0% after treatment with 3% NaOH. FTIR analysis supported these observations at the functional group level, revealing a decrease in absorbance at 1510 cm^−1^ and 1240 cm^−1^ by 89% and 97%, respectively, following alkali treatment. Furthermore, the A_1240_/A_1730_ ratio significantly decreased by 77%, suggesting effective disruption of lignin-related linkages under alkaline conditions. Compared with the 39% lignin removal reported by Ni (2010) for alkali-treated *Achnatherum splendens*, our study showed a lower lignin dissolution rate, which may be related to differences in raw material lignin content (16.29% vs. 9.59%). In comparison to other grass materials, Gao et al. (2020b) reported that Pennisetum hybrid achieved an 88% lignin removal rate under conditions of 3% NaOH, 120 °C, and 15 min. Xu and Cheng (2011) reported that switchgrass reached approximately 62% lignin removal under 2% NaOH, 121 °C, and 30 min, both of which are significantly higher than the 30.3% removal rate observed for *Achnatherum splendens* in this study. Recent studies have further confirmed the high efficiency of alkaline treatment in delignification. Zhang et al. (2025a) found that sunflower stem bark achieved an enzymatic hydrolysis yield of 96.7% following NaOH pretreatment, which significantly outperformed the 26.8% yield from dilute acid treatment. Kaur et al. (2025) demonstrated that treating sugarcane bagasse with 1 M NaOH for 60 min resulted in an 83.17% lignin removal, whereas acid treatment exhibited almost no significant delignification effect. Furthermore, Premjet and Premjet (2025) revealed that the pretreatment of brome grass with 2% NaOH at 120 °C achieved a 74.7% lignin removal and a 93.2% glucan recovery. SEM observations provided visual morphological evidence for the effective deconstruction resulting from alkaline treatment. Following treatment with 3% NaOH, the inner surface fiber bundles exhibited distinct fibrillation phenomena, with the fiber surfaces displaying tearing and branching characteristics. Microfibrils became exposed and formed filamentous connections. The outer surface coating was entirely removed, allowing the fiber bundles to separate fully into individual fibers. These fibers interwove and twisted with each other, forming an irregular network structure, while the fiber diameter significantly decreased and exhibited swelling phenomena. Similar observations of loosened fiber bundles and a cleaned surface morphology were reported by Gao et al. (2022) in the study of NaOH-pretreated hybrid Pennisetum. These morphological changes were consistent with lignin removal. As the 'adhesive' in cell walls, lignin removal results in a loss of bonding force between cellulose microfibrils, thereby facilitating thorough fiber bundle dissociation. It is noteworthy that, although the fiber bundles exposed after acid treatment still show residual attachments, the alkali-treated fibers exhibit a loosened structure with clean surfaces. This morphological difference supports the distinction between the two pretreatment methods regarding lignin removal efficiency.

#### Anomalous component degradation and possible mechanistic explanations

An anomalous phenomenon was observed in the single-factor experiment involving dilute sulfuric acid concentration: the cellulose degradation rate under 0.5% H_2_SO_4_ treatment (16.8%) was significantly higher than that of the 1.0–2.0% treatment groups (12–14%) (*P* < 0.05). Theoretically, the cellulose degradation rate should increase with higher acid concentration; however, in this study, treatment with a lower acid concentration unexpectedly resulted in greater cellulose loss. This phenomenon may be attributed to two potential reasons: First, under conditions of extremely low acid concentration, H₃O⁺ may not sufficiently act on the hemicellulose matrix, but instead preferentially attacks the β-1,4-glycosidic bonds exposed in the amorphous regions of cellulose or on microfibril surfaces. Second, limited hemicellulose dissolution following low-concentration acid treatment may result in insufficient development of cell wall pores, potentially causing partial mechanical detachment of cellulose fragments during subsequent washing due to physical shear forces. Since this study did not conduct monosaccharide analysis of degradation products, the above hypotheses require further verification. Nonetheless, this phenomenon suggests that during the optimization of dilute acid pretreatment processes, excessively low acid concentrations may not only reduce hemicellulose removal efficiency but also lead to unnecessary cellulose loss.

In the single-factor experiment examining the NaOH pretreatment time, both the lignin dissolution rate and cellulose degradation rate exhibited nonlinear changes. Specifically, when the treatment time was extended from 15 to 45 min, the lignin dissolution rate significantly increased from 22.6 to 49.0% (*P* < 0.05), while the cellulose degradation rate decreased from 19.7 to 15.3%. However, when the treatment time was further prolonged to 90 min, the lignin dissolution rate dropped to 30.6%, whereas the cellulose degradation rate rebounded to 24.8%. This nonmonotonic trend may be associated with two parallel chemical processes: when the treatment duration is excessively long (≥ 90 min), dissolved lignin fragments may undergo recondensation or redeposition under high-temperature alkaline conditions, reattaching to the fiber surface and resulting in a decreased apparent lignin dissolution rate. Simultaneously, the reducing ends of cellulose may undergo peeling reactions in the high-temperature alkaline environment, causing layer-by-layer exfoliation of cellulose crystal surfaces and consequently increasing the degradation rate. Similar phenomena have been reported in the alkaline pretreatment of straw-based feedstocks (Kim and Lee 2007; Zheng et al. 2012). This finding provides a preliminary reference for selecting the pretreatment duration for *Achnatherum splendens*: a treatment time of 45–60 min appears to balance lignin removal and cellulose retention under the single-factor conditions used in this study.

In summary, the pretreatment of dilute sulfuric acid effectively removes hemicellulose while causing limited delignification, primarily by creating porous channels in the cell walls through the hydrolysis of hemicellulose. However, the presence of residual lignin and possible pseudo-lignin-like deposits may hinder complete separation of fiber bundles. In contrast, sodium hydroxide pretreatment demonstrates effective delignification alongside the dissolution of hemicellulose, resulting in thorough separation of fiber bundles and surface purification by completely disrupting the structures of the cell walls. These two pretreatment methods exhibit complementary effects. Given that *Achnatherum splendens* possesses a relatively high lignin content and shows a weaker response to alkaline treatment, this indicates the necessity for stronger pretreatment conditions or the implementation of combined treatment strategies.

#### Preliminary process windows based on single-factor experiments

Based on the single-factor results, dilute sulfuric acid pretreatment showed a preliminary effective window at approximately 1.5% H_2_SO_4_, a solid-to-liquid ratio of 1:10–1:14, a treatment time of 30–60 min, and a temperature of 120 °C. Under these conditions, hemicellulose degradation reached approximately 79–89%, cellulose degradation remained below 15%, and lignin dissolution ranged from 20 to 31%. However, this range should not be regarded as a fully optimized pretreatment condition. Instead, it represents a preliminary process window derived from single-factor main effects.

For NaOH pretreatment, the preliminary effective window was approximately 3–4% NaOH, a solid-to-liquid ratio of 1:10–1:14, a treatment time of 45–60 min, and a temperature range of 110–120 °C. Within this range, lignin dissolution reached approximately 45–52%, hemicellulose degradation ranged from 65 to 75%, and cellulose degradation remained between 15 and 30%. Similarly, this range should be interpreted as a single-factor-based screening result rather than an optimized process condition, because interactions among NaOH concentration, solid-to-liquid ratio, treatment time, and temperature were not evaluated in this study. Further multivariate optimization would be required before these conditions can be considered optimized for downstream application.

### Effects of pretreatment on cellulose crystalline structure

The XRD analysis results indicate that both dilute sulfuric acid and sodium hydroxide pretreatments significantly enhanced the cellulose crystallinity of treated samples compared to the raw material, while the crystallite size exhibited no notable change. The crystallinity of untreated *Achnatherum splendens* was measured at 47.0%, which increased to 57.2% following acid treatment and to 55.0% after alkali treatment. This increase in crystallinity may be primarily attributed to the preferential removal of amorphous components—including hemicellulose, lignin, and amorphous regions of cellulose—through acid–base pretreatment, which passively increases the relative proportion of crystalline cellulose (DeMartini et al. 2013; Kapoor et al. 2015). Recent studies have corroborated this mechanism of "preferential removal of amorphous components leading to a passive increase in crystallinity.” For instance, Hossen et al. (2024) observed a marked increase in crystallinity during the preparation of nanocellulose via multi-step chemical treatments, including alkali treatment, bleaching, and acid hydrolysis, which was linked to the effective removal of amorphous regions. Wakudkar et al. (2025) reported that the crystallinity of cellulose extracted from corn cobs increased from 29.63 to 53.95% after alkali pretreatment and bleaching. Similarly, Hasan et al. (2025) found that the crystallinity reached approximately 62.5% after alkali extraction and acid hydrolysis of cellulose nanocrystals (CNC) from jute fibers, supporting the relationship between amorphous component removal and increased relative crystallinity. Notably, acid treatment primarily enhances crystallinity by dissolving a substantial portion of hemicellulose (79–89%), whereas alkali treatment mainly achieves this through lignin removal (45–52%). However, the higher cellulose loss rate (15–30%) during alkali treatment partially mitigates the crystallinity improvement effect resulting from the removal of amorphous components.

The increase in crystallinity is a common phenomenon associated with chemical pretreatment. Gao et al. (2020a) demonstrated that the crystallinity of hybrid Pennisetum increased from 33.0 to 43.8% following acid treatment. A similar trend was observed by Li et al. (2016) in radiation pretreatment experiments involving Arundo donax and Miscanthus. Notably, Sethi et al. (2025) reported that alkali-assisted photocatalytic pretreatment altered the crystalline characteristics of rice straw, suggesting that pretreatment intensity can influence cellulose crystallinity. Moderate pretreatment may increase the apparent crystallinity by removing amorphous components, whereas excessive treatment may partially disrupt crystalline cellulose regions. In this study, the increase in crystallinity of *Achnatherum splendens* was within a comparable range to the results reported in the aforementioned literature. However, due to the initially high crystallinity of the raw material, it retained a relatively high level of crystallinity post-treatment.

The untreated *Achnatherum splendens* exhibited a crystallite size of 2.99 nm, whereas the pretreated samples displayed crystallite size ranging from 3.05 to 3.32 nm. Statistical analysis revealed no significant difference (*P* > 0.05). These findings indicate that, under the experimental conditions of this study, the acid–base treatment primarily affected the hemicellulose and lignin in the non-crystalline regions and microfibril surfaces, without disrupting the ordered structure within the crystalline regions. Recent studies have further corroborated this pattern: Kar et al. (2025) reported that a 7.5% NaOH treatment significantly increased crystallinity, yielding a crystallite size of 3.03 nm, while a 4 wt.% NaOH treatment similarly enhanced crystallinity with a crystallite size of 2.64 nm. These values closely align with the range observed in this study (2.99–3.32 nm), providing additional evidence that alkali treatment improves crystallinity without significantly affecting crystallite size. In contrast, Zhang et al. (2025b) noted the destruction and swelling of the cellulose crystalline structure during alkali-assisted ball milling pretreatment, with significant changes in crystallite size, which sharply contrasts with the findings of this study. This suggests that the relatively mild alkaline treatment conditions applied in this study (120 °C, 30 min, without mechanical assistance) were insufficient to disrupt the internal structure of crystalline regions, whereas more severe treatments (such as ball milling) could further compromise the crystalline structure, thereby enhancing cellulose accessibility. Because instrumental broadening was not corrected in this study, crystallite size values should be interpreted as apparent values for relative comparison rather than absolute crystal dimensions.

All NaOH-pretreated samples exhibited a distinct diffraction peak at approximately 2θ ≈ 29.5° in the XRD patterns, which was absent in both the raw materials and acid-treated samples. This diffraction peak may be associated with sodium carbonate or sodium carbonate hydrate residues formed by the reaction of residual NaOH with atmospheric CO_2_ during post-treatment handling (Dickens et al. 1970; Harabor et al. 2013). The position of this peak lies outside the primary diffraction region of cellulose, and after background subtraction, it does not influence the calculated results of crystallinity and crystallite size.

### FTIR anomalies and possible pseudo-lignin-related interference

There exists a discrepancy between the FTIR analysis results and the degradation rates determined by chemical methods, a phenomenon particularly evident in samples pretreated with dilute sulfuric acid. Chemical analysis indicated that after treatment with 1.5% H_2_SO_4_, the degradation rate of *Achnatherum splendens* hemicellulose reached 79.2%, while the dissolution rate of lignin was only 20.5%. According to the principles of FTIR semi-quantitative analysis, the absolute absorbance of the hemicellulose acetyl characteristic peak at 1730 cm^−1^ and the lignin aromatic ring characteristic peak at 1510 cm^−1^ would generally be expected to decrease after component removal. However, the FTIR results showed three anomalies: (1) The absorbance at 1510 cm^−1^ not only failed to decrease but increased from 3.824 to 4.009, representing an approximately 5% increase, which contradicts the lignin retention result (20.5% dissolution rate) obtained from chemical analysis; (2) The absorbance at 1730 cm^−1^ decreased by only 11% (from 4.137 to 3.676), significantly lower than the expected reduction corresponding to the actual 79% hemicellulose degradation rate; (3) The A_1240_/A_1730_ ratio abnormally increased from 0.989 to 1.181 (a 19% increase), indicating a significant alteration in the proportional relationship between carbonyl absorption at 1730 cm^−1^ and C–O absorption at 1240 cm^−1^. These anomalous phenomena cannot be explained by variations in natural components, suggesting the possible generation, during the acid treatment process, of pseudo-lignin-like substances that may exhibit infrared absorption characteristics near both 1510 cm^−1^ and 1730 cm^−1^. Chen et al. (2024) noted in a review that pseudo-lignin is identified as Klason lignin in compositional analysis, while in Py-GC–MS analysis it is recognized as aromatic compounds derived from carbohydrates. Sheng et al. (2021) further demonstrated that pseudo-lignin formed during dilute acid pretreatment significantly inhibits the enzymatic hydrolysis process.

Pseudo-lignin has been reported to form under acidic pretreatment conditions through condensation reactions involving carbohydrate-derived intermediates, such as furfural and 5-hydroxymethylfurfural, with lignin fragments or other degradation products (Sannigrahi et al. 2011; Hu et al. 2012). Such materials may contain aromatic, furan, phenolic, and carbonyl-containing structures, which can contribute to absorption near 1510 cm^−1^ and 1730 cm^−1^ (Chen et al. 2024; Wang et al. 2024). Therefore, the increased absorbance at 1510 cm^−1^ in acid-treated samples may be related to additional aromatic absorption from pseudo-lignin-like structures, while the limited decrease at 1730 cm^−1^ may be affected by overlapping carbonyl absorption. The increase in the A_1240_/A_1730_ ratio may also reflect changes in the relative contributions of C–O and C=O absorption after acid treatment. In contrast, NaOH pretreatment caused clear decreases in the absorbance at 1510 cm^−1^ and 1730 cm^−1^, which is consistent with stronger delignification and reduced interference from acid-derived condensation products.

These results suggest that pseudo-lignin-related interference may affect the semi-quantitative interpretation of FTIR spectra in dilute acid pretreatment systems. In such cases, FTIR is useful for tracking functional group changes, but component degradation should be interpreted together with chemical composition analysis. The infrared crystallinity index A_1425_/A_898_ should also be used cautiously because it may be affected by multiple factors, including exposure of amorphous cellulose, C–O absorption from pseudo-lignin-like structures, and residual inorganic substances after alkali treatment. Therefore, XRD provides more reliable information for evaluating cellulose crystal structure (Kruer-Zerhusen et al. 2018).

Overall, the FTIR anomalies observed in acid-treated *Achnatherum splendens* may be associated with pseudo-lignin-related interference. However, pseudo-lignin was not directly isolated or structurally confirmed in this study; therefore, this interpretation should be regarded as a possible explanation rather than direct confirmation. In addition, although pseudo-lignin has been reported to create non-productive adsorption sites and physical barriers for cellulase, its actual effect on enzymatic hydrolysis of pretreated *Achnatherum splendens* was not tested in the present study (Hu et al. 2012; Li et al. 2014; Chen et al. 2024). Future studies using Py-GC/MS, XPS, or 2D-HSQC NMR are needed to verify the formation and structural characteristics of pseudo-lignin in this system.

### Application implications of pretreatment strategies

The results of this study provide structural-level guidance for selecting pretreatment strategies for high-lignin energy grasses such as *Achnatherum splendens*. Dilute acid pretreatment was effective in removing hemicellulose and opening the cell wall structure, but its limited delignification ability suggests that it may not be sufficient as a standalone pretreatment for feedstocks with relatively high lignin content. In contrast, NaOH pretreatment showed stronger lignin removal and fiber separation, which may be beneficial for exposing cellulose-rich fractions. Pretreatment-derived cellulose-rich fractions may also have potential for further material-oriented valorization (Do et al. 2025).

From a practical perspective, acid and alkali pretreatments may play complementary roles. Dilute acid treatment could serve as a preliminary step for hemicellulose removal and pore formation, whereas alkali treatment could further reduce lignin barriers and promote fiber separation. Therefore, a sequential or combined acid–alkali pretreatment strategy may be worth further investigation for *Achnatherum splendens* and other high-lignin herbaceous biomass.

Nevertheless, the application implications proposed here are based on chemical composition and structural characterization rather than direct conversion experiments. Because enzymatic hydrolysis was not performed in this study, the present results cannot directly demonstrate improved sugar yield or enzymatic digestibility. Future enzymatic saccharification experiments are required to evaluate whether the observed structural changes can improve downstream conversion performance.

### Limitations and future research perspectives

This study investigated the effects of acid and alkali pretreatments on the composition and structure of *Achnatherum splendens* using multi-scale characterization techniques, and provided possible FTIR-based evidence of pseudo-lignin-related interference. Nevertheless, several limitations persist. Firstly, the study did not conduct enzymatic hydrolysis experiments on the solid residues post-pretreatment, and therefore could not directly assess whether pretreatment improved cellulose accessibility or quantify the actual inhibitory impact of pseudo-lignin on enzymatic hydrolysis (Hu et al. 2012; Li et al. 2014). In addition, monosaccharide analysis of degradation products was not performed, so the proposed explanation for abnormal cellulose loss under low acid concentration requires further verification. Recent research has confirmed the significant inhibitory effect of pseudo-lignin on enzymatic hydrolysis (Wang et al. 2024; Chen et al. 2024). Secondly, there was no quantitative analysis of the dissolved sugars and potential inhibitors (including furfural, HMF, and acetic acid) in the pretreatment liquid, which resulted in a lack of evaluation regarding the hemicellulose recovery value and the risks posed by inhibitors (Palmqvist and Hahn-Hägerdal 2000). Thirdly, the study exclusively compared single-reagent pretreatments using dilute sulfuric acid and NaOH, without investigating the potential of combined acid–base pretreatment. Existing literature indicates that sequential acid and alkali pretreatment can significantly enhance cellulose content (Cui et al. 2017; Ni 2010). Fourth, the single-factor experimental design employed in this study fails to assess interactions among various factors; thus, response surface methodology could be employed to further optimize the process. Fifth, while FTIR anomalies suggested possible pseudo-lignin-related interference, pseudo-lignin was not directly isolated or structurally confirmed. Lastly, the study focused solely on *Achnatherum splendens* cultivated at the Beijing Xiaotangshan base; therefore, the generalizability of the findings to raw materials from diverse origins necessitates further validation.

Based on the aforementioned limitations, subsequent research could be conducted in several directions. First, enzymatic saccharification experiments should be performed to measure the conversion rates of glucose and xylose, thereby directly evaluating the effectiveness of the pretreatment process. Second, techniques such as Py-GC/MS and 2D-HSQC NMR should be employed to confirm the chemical structure of pseudo-lignin and quantitatively assess its inhibitory mechanisms on cellulase activity. Third, an acid–alkali combined pretreatment process should be designed to investigate the sequencing and parameters of the two steps, utilizing global optimization through response surface methodology. Additionally, strategies such as the incorporation of histidine to reduce pseudo-lignin formation could be explored. Fourth, samples of *Achnatherum splendens* from various regions should be collected to establish predictive models that correlate feedstock characteristics with pretreatment efficacy. Furthermore, monosaccharides and inhibitors in the pretreatment liquid should be analyzed to explore resource utilization pathways for xylose conversion and lignin recovery. Lastly, other pretreatment methods, such as deep eutectic solvents and steam explosion, should also be investigated to identify efficient and cost-effective technical routes suitable for *Achnatherum splendens*.

## Conclusion

This study compared dilute sulfuric acid and sodium hydroxide pretreatments for cell wall deconstruction of *Achnatherum splendens* using chemical composition analysis, XRD, FTIR, and SEM. Dilute sulfuric acid pretreatment mainly promoted hemicellulose degradation, whereas NaOH pretreatment showed stronger effects on lignin removal and fiber separation. Both pretreatments increased cellulose crystallinity while maintaining relatively stable crystallite size, indicating that the crystalline cellulose domains were largely preserved. FTIR results suggested possible pseudo-lignin-related interference under acid pretreatment, but direct structural confirmation was not performed. The single-factor results indicate that acid and alkali pretreatments have complementary effects on the deconstruction of high-lignin *Achnatherum splendens*. These findings provide structural-level evidence for selecting pretreatment strategies for high-lignin *Achnatherum splendens*. Future enzymatic hydrolysis and degradation product analyses are required to evaluate the conversion performance of the proposed pretreatment strategies.

## Data Availability

The datasets used and analyzed during the current study are available from the corresponding author on reasonable request.
